# Distinct expression of NEAT1 isoforms in Parkinson’s disease models suggests different roles of the variants during the disease course

**DOI:** 10.1038/s41598-025-95787-0

**Published:** 2025-04-15

**Authors:** Fanni Annamária Boros, Orsolya Horváth, Rita Maszlag-Török, Mária Baranyi, Nikolett Nánási, Orsolya Oláh-Németh, Beáta Sperlágh, László Vécsei, Péter Klivényi

**Affiliations:** 1 Department of Neurology, Albert Szent-Györgyi Clinical Center, Faculty of Medicine, University of Szeged, Szeged, Hungary; 2 HUN-REN-SZTE Functional Clinical Genetics Research Group, Hungarian Research Network, Szeged, Hungary; 3 Doctoral School of Clinical Medicine, University of Szeged, Korányi Fasor, 66720 Szeged, Hungary; 4 Laboratory of Molecular Pharmacology, HUN-REN Institute of Experimental Medicine, Budapest, Hungary; 5 Department of Pathology, School of Medicine, University of Szeged, Szeged, Hungary; 6 HUN-REN-SZTE Neuroscience Research Group, Szeged, Hungary; 7 Present Address: Department of Molecular Neurology, University Hospital Erlangen, Friedrich-Alexander University Erlangen-Nürnberg, 91054 Erlangen, Germany

**Keywords:** Parkinson’s disease, NEAT1, Neat1, lncRNA, MPTP, Neurodegeneration, MAO, Cell biology, Neuroscience

## Abstract

Parkinson’s disease (PD) is the second most common neurodegenerative disease worldwide. Recently long non-coding RNAs (lncRNAs) have emerged as possible molecular hubs in the diverse pathomechanisms of the disease. Among them, NEAT1 gained particular interest due to findings suggesting both protective and deleterious effects of this lncRNA in PD models.The aim of this study was to clarify some of the contradictions among data that appeared in recent publications concerning NEAT1 effects. For this, we determined whether pharmacological increase of NEAT1 levels worsened the detrimental effect of MPP + in the SH-SY5Y cell model, and whether the levels of the short and long isoform of the lncRNA changed differently upon short and extended MPTP treatment in an MPTP-induced mouse model of PD. Our findings suggest differential expression of NEAT1/Neat1 isoforms in MPP + /MPTP-induced PD models, which is in accord with the proposed role of the lncRNA in the general stress response. We propose that first an early up-regulation of Neat1_2 is dominant. The level of Neat1_2 then decreases as pathology progresses, resulting in a shift in the ratio of the two isoforms towards a higher level of Neat1_1 accompanied by damage of the central nervous system.

## Introduction

Despite Parkinson’s disease (PD) being the second most common neurodegenerative disease and one of the leading causes of disability worldwide^1^, to date there is no curative therapy or early disease biomarker available, partially due to our incomplete understanding of the pathomechanism of the disease. PD is characterized by loss of the dopaminergic neurons in the substantia nigra; however, the reason for the selective loss of these cells is not fully understood^2^.

Intensive research has been undertaken to elucidate the molecular pathomechanisms of PD. As a result, alterations in various genes and impaired function of a wide range of molecules have been identified. Recently long non-coding RNAs (lncRNAs), and among them, Nuclear Enriched Abundant Transcript 1 (NEAT1) has emerged as a possible molecular crossroad at which several mechanisms related to neurodegeneration may interconnect^3^.

The *NEAT1*/*Neat1* gene is located on chromosome 11 and 19 in human and mouse, respectively. Transcription by RNA polymerase II and RNA processing give rise to two major isoforms from this locus. The human short isoform (NEAT1_1) is 3.7 kb, while the long (NEAT1_2) spans 23 kb. In mice the short and long products, Neat1_1 and Neat1_2 are 3.1 kb and 20 kb, respectively^3^. The two isoforms are identical at their 5’ end, making the separate detection of NEAT1_1/Neat1_1 and NEAT1_2/Neat1_2 a challenging task.

Besides size and structure, the two NEAT1 isoforms also differ in their functions. NEAT1_2 is an essential component of paraspeckles, 0.2–1 μm cell organelles in the interchromatin region of the mammalian nucleus^4–6^. It has been found that the basal number of paraspeckles increases upon various cellular stresses such as mitochondrial stress, hypoxia and heat shock, contributing to a general cytoprotective process. Paraspeckles regulate gene expression both directly and indirectly by various means: they are involved in transcription regulation through transcription factor sequestration, in translation regulation via the nuclear retention of A-to-I hyperedited RNAs and in pri-miRNA processing modulation (reviewed in^3^). NEAT1_2 serves as a scaffold for paraspeckle proteins, including several regulators of transcription and RNA splicing^7^.

While the cardinal role of NEAT1_2 in paraspeckle formation is generally acknowledged, the cellular localisation and function of NEAT1_1 is still debated. There are findings on NEAT1_1 co-localisation with NEAT1_2 in paraspeckles, and on the short isoform residing outside of paraspeckles, in so called microspeckles^8^.

Alteration in the levels of NEAT1 has been reported in various malignant diseases (for a review see^9^), and findings of a growing number of studies indicate opposing effects of the long and short isoforms. While knockdown of NEAT1_1 in colorectal cancer cell lines attenuated the invasive capacity and viability of the cells, NEAT1_2 down-regulation resulted in increased cell growth. NEAT1_2 and NEAT1_1 have also been found to have opposite effects in the context of neuroblastoma: the short isoform was found to promote cell proliferation, whereas the long isoform exerted a tumour-suppressive role^10^.

Similarly, NEAT1 has been proposed to have a dual role in the motoneuron disease amyotrophic lateral sclerosis (ALS). High NEAT1_2 levels have been shown in ventral horn motoneurons in early disease stages; however, as the disease progresses, NEAT1_2 levels gradually decrease^11–14^. More recently, NEAT1 has been identified as a genetic modifier of ALS onset^15^.

In regard to the role of NEAT1 in PD and PD models, published data are conflicting. Some articles argue for a protective role based on findings that NEAT1 overexpression leads to increased cell viability. This effect is attributed mainly to the elevated level of the long isoform^16^. In contrast, several publications report that silencing of NEAT1 increases cell viability and protects against the loss of dopaminergic neurons in *in vivo* models of PD (for a review see^17^).

In most of the reports showing effects of NEAT1 level modulation however, it is not specified whether the up/down-regulation of the short or the long isoform was achieved. Notably, while the majority of papers attribute adverse effects to NEAT1 (without specification of the short and long isoform), the few which distinguish NEAT1_2 overproduction find it not to be detrimental.

With the intention to clarify some of the contradictions regarding the role of NEAT1 in PD, we aimed to determine whether pharmacological increase of NEAT1 level increases the detrimental effect of 1-methyl-4-phenylpyridinium (MPP +) in an SH-SY5Y cell model of the disease. Furthermore, we sought to ascertain whether there is any difference in the Neat1 response upon 1-methyl-4-phenyl-1,2,3,6-tetrahydropyridine (MPTP) treatment in a mouse PD model in respect of the two isoforms of the lncRNA.

## Results

### SH-SY5Y cells respond with increased NEAT1 expression to both MPP + and SFN but the two drugs affect cell viability differently

Based mostly on a rescue effect of siRNA-based down-regulation of NEAT1, numerous studies attribute an adverse effect to increased NEAT1 levels on cell viability upon MPP + toxin administration in the SH-SY5Y cell model of PD. In contrast, other studies found an increased NEAT1 level to be beneficial to cell survival. We sought to help clarify these conflicting findings by increasing the level of the lncRNA via pharmacological activation of the *NEAT1* gene.

*NEAT1* has been identified as a target of heat shock factor 1 (HSF1)^18^. Upon activation of the heat shock (HS) pathway, HSF1 binds to a heat shock element within the *NEAT1* promoter region and enhances the expression of the lncRNA. In accord with this, sulforaphane (SFN), a natural isothiocyanate that is an activator of HSF1, has been recently described to cause NEAT1 up-regulation in HeLa cells^18^.

**Fig. 1:**
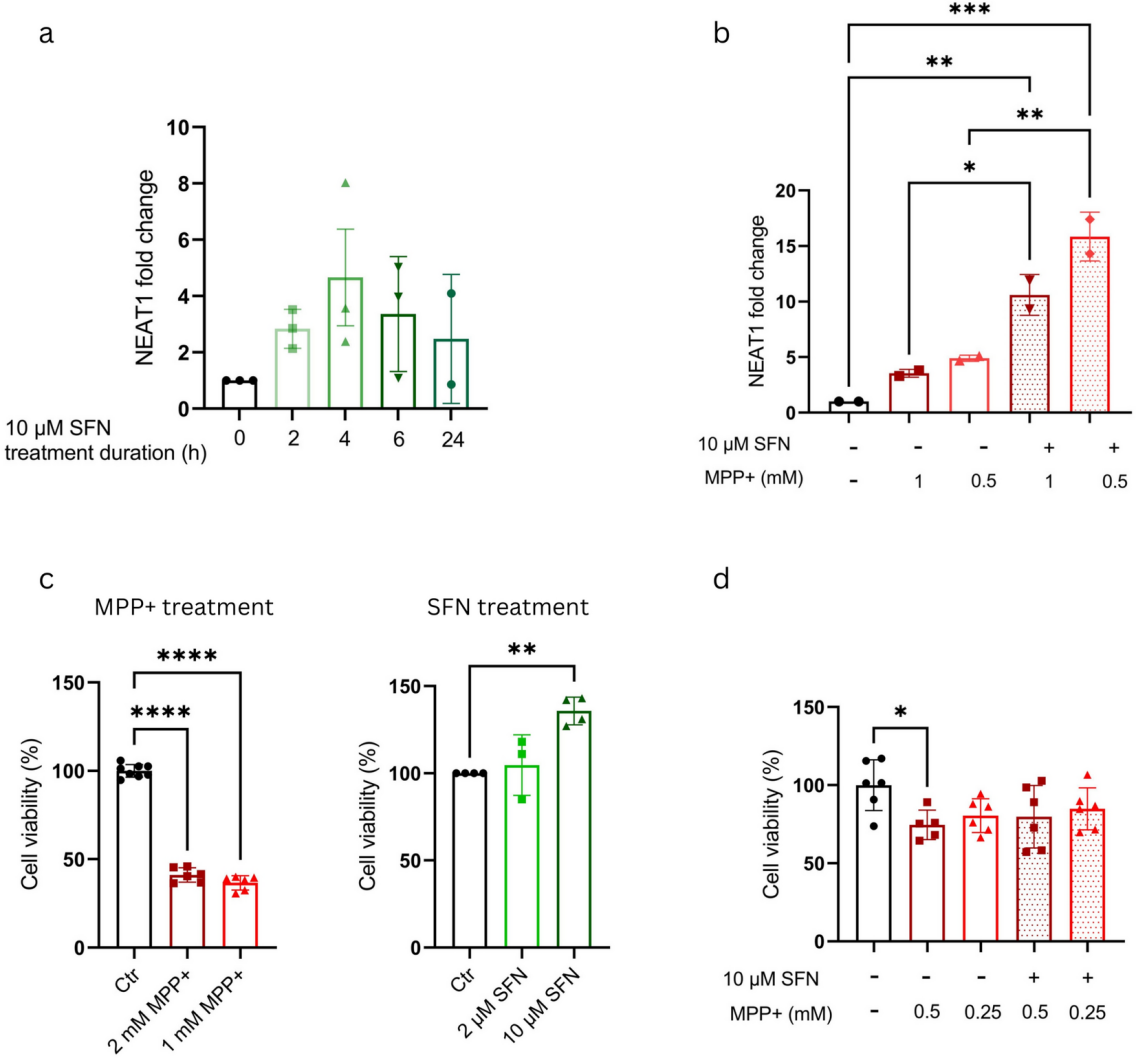
NEAT1 expression and viability in/of SH-SY5Y cells upon combined MPP + and SFN treatment. (**a**) SFN increases NEAT1 expression in SH-SY5Y cells. (**b**) MPP + and SFN have additive effects on NEAT1 up-regulation in SH-SY5Y cells. NEAT1 RNA level was determined by quantitative RT-PCR as described in Methods. (**c**) MPP + has a strong toxic effect and decreases cell viability, while SFN treatment does not decrease, but rather increases cell viability. (**d**) SFN does not enhance the toxic effects of MPP + , although it is unable to reverse the cell viability decrease caused by toxin treatment. Viability of SH-SY5Y cells was determined after 24 h of culturing in the presence of the indicated drug(s), at the concentrations shown, by the use of CKK-8 assay. For testing data distribution the Shapiro–Wilk test was used, followed by one-way ANOVA and Dunnett’s multiple comparison test (n = 2–6). Values are plotted as mean ± SD. Datapoints represent values detected for individual replicates or the average of those from multiple independent experiments (panel c right graph). *NEAT1* Nuclear enriched abundant transcript 1, *SFN* sulforaphane, *MPP* + *1*-methyl-4-phenylpyridinium.

We found that in SH-SY5Y cells SFN treatment also results in an increase in NEAT1 level (Fig. 1a). Furthermore, a combined treatment with SFN and MPP + resulted in an additive effect on NEAT1 level (Fig. 1b).

The verification that SFN is an inducer of *NEAT1* expression opened up the possibility of investigating the effect of NEAT1 overproduction on cell viability. We found that although SFN acts as an inducer of *NEAT1* similarly to MPP + , SFN treatment of SH-SY5Y cells resulted in an increase rather than a decrease in cell viability. (Fig. 1c). This in contrast with the effects of MPP + , which causes a significant reduction in cell viability, as also reported previously (Fig. 1c). In combination with MPP + , SFN did not enhance the toxic effects of the drug (Fig. 1d). Moreover, SFN treatment had a rescue effect on the MPP + treatment-induced apoptosis of SH-SY5Y cells. Annexin V-FITC fluorescence-activated sorting of cells treated with 0.25 mM or 0.5 mM MPP + in combination with 10 µM SFN for 24 h revealed a smaller fraction of cells displaying signs of apoptosis than analyses of cells treated with MPP + only (Supplementary Fig. 1.).

### SFN alone has a weak effect on *in vivo Neat1* expression and might act as a co-inducer in combination with MPTP

To evaluate whether SFN is also suitable for the modulation of *in vivo Neat1* expression, we treated mice with different doses (D1 = 2.5, D2 = 5, and D3 = 10 mg/kg of body weight) of SFN dissolved in EtOH, and determined the changes in the total Neat1 and Neat1_2 levels in the brainstem, striatum, cortex, and cerebellum 1.5, 6, 12, and 24 h following treatment. Controls in these experiments received intraperitoneal (i.p.) injections of 14.1% EtOH diluted in 0.9% NaCl solution, the vehicle of SFN. Neat1 and Neat1_2 levels were determined by quantitative RT-PCR using primers and reaction conditions given in Table 1. and Table 2. in the Methods section.

**Table 1:** Primer sequences used for *in vitro* and *in vivo* analyses.

*In vitro* experiments		
NEAT1	Fw	GGGCCATCAGCTTTGAATAA
	Rev	GGTGGGTAGGTGAGAGGTCA
18S	Fw	GCTTAATTTGACTCAACACGGGA
	Rev	AGCTATCAATCTGTCAATCCTGTC

*In vivo* experiments		
Neat1	Fw	TTGGGACAGTGGACGTGTGG
	Rev	TCAAGTGCCAGCAGACAGCA
Neat1_2	Fw	GCTCTGGGACCTTCGTGACTCT
	Rev	CTGCCTTGGCTTGGAAATGTAA

*NEAT1/Neat1* Nuclear enriched abundant transcript 1, *Fw* forward, *Rev* reverse.

**Table 2:** Cycling conditions of RT-qPCR reactions.

*In vitro* experiments			
Gene	Temperature	Time	Number of cycles
NEAT1 and 18S	50 °C	2 min	1
	95 °C	2 min	1
	95 °C	30 s	40
	57 °C	45 s	
	72 °C	30 s	

*In vivo* experiments			
Gene	Temperature	Time	Number of cycles
Neat1 and Neat1_2	50 °C	2 min	1
	95 °C	2 min	1
	95 °C	30 s	40
	63 °C	45 s	
	72 °C	30 s	
18S	95 °C	10 min	1
	95 °C	15 s	40
	60 °C	1 min	

*NEAT1/Neat1* Nuclear enriched abundant transcript 1, *Fw* forward, *Rev* reverse.

SFN administration did not result in a large increase in the level of Neat1 in any of the investigated brain regions at any of the tested concentrations (Supplementary Fig. 2 a, c, e, and g). Nonetheless, a slight but significant increase in Neat1_2 level was observed in brainstem samples of animals treated with the higher doses at 6 h (Supplementary Fig. 2. b). A similar trend was observed in striatal samples following 12 h treatment; however, this difference did not prove to be significant (Supplementary Fig. 2. d). Similarly, no significant up-regulation was observed in cortex and cerebellum samples (Supplementary Fig. 2. f and h). Surprisingly, the lowest dose of SFN in brainstem-6 h samples resulted in a decrease of Neat1 level (Supplementary Fig. 2. a).

**Fig. 2:**
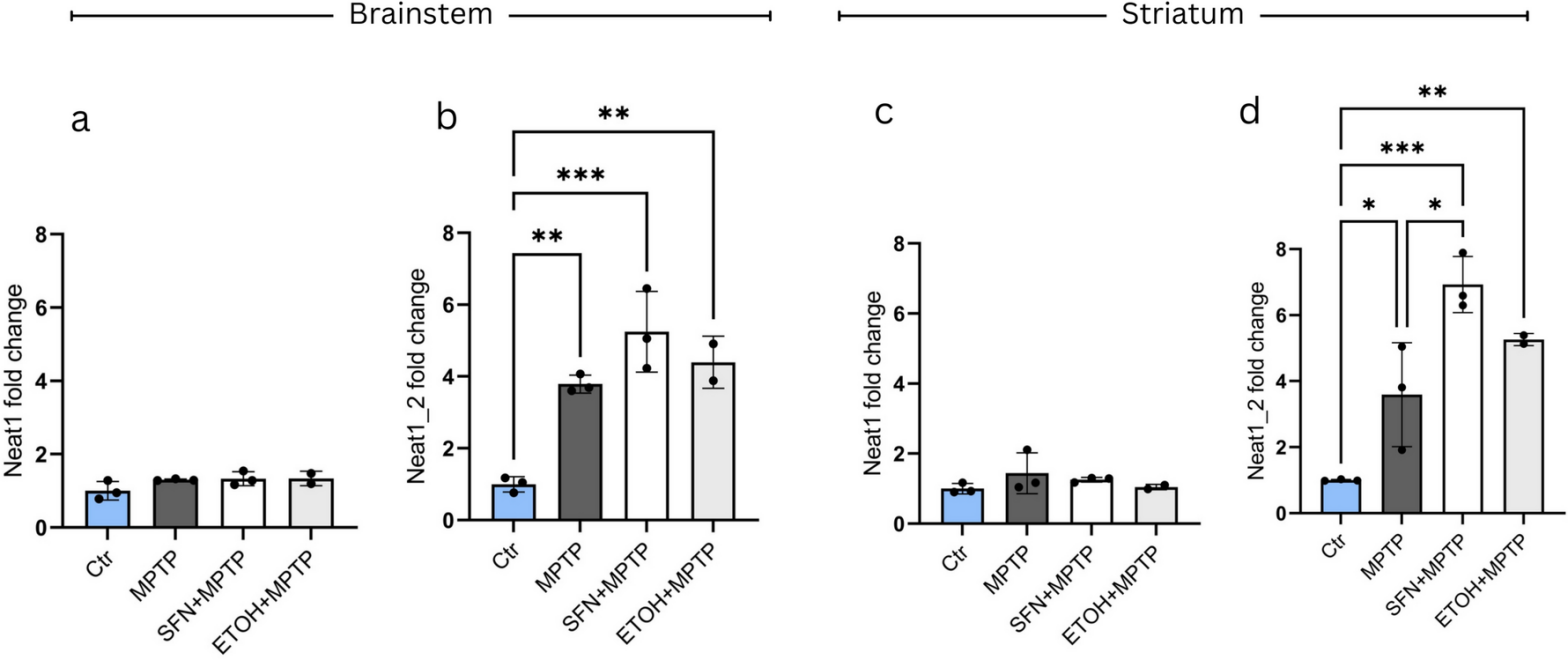
*In vivo *effects of co-treatment with MPTP and SFN on NEAT1 expression. MPTP does not result in significant up-regulation of the total Neat1 level in brainstem and striatum samples of mice, either alone, or in combination with SFN or its vehicle, EtOH (**a** and **c**) Analysis of the level of the long isoform Neat1_2, however, revealed significant increase upon MPTP treatment both in brainstem (**b**) and striatal samples (d) (*p* = 0.004 and 0.0497, respectively). Combined treatment with SFN and MPTP results in an even more prominent increase in the expression of Neat1_2 compared to the control (Ctr) in both investigated brain regions (brainstem: *p* = 0.003; striatum: *p* = 0.0006). (Comparisons of MPTP, SFN + MPTP and EtOH + MPTP indicate that EtOH modifies the effect of MPTP on Neat1 expression under the conditions used, limiting comparisons for MPTP and SFN + MPTP effects). For the assessment of data distribution the Shapiro–Wilk test was used, followed by one-way ANOVA. For multiple comparisons the Tukey’s Multiple comparison test was implemented (n = 2–3). Values are plotted as mean ± SD. Datapoints represent values detected in individual animals. *Ctr* control, *MPTP* 1-methyl-4-phenyl-1,2,3,6-tetrahydropyridine* ,NEAT1* Nuclear enriched abundant transcript 1, *SFN* sulforaphane, *MPP* + *1*-methyl-4-phenylpyridinium.

To determine if co-treatment with SFN and MPTP toxin has additive effects on enhancing Neat1 expression *in vivo*, we next compared Neat1 levels in striatum or brainstem samples of MPTP treated and SFN + MPTP treated animals. Animals of the former group received 15 mg/kg of body weight toxin injections 3 times, with 2 h between doses. MPTP was dissolved in PBS buffer and animals of the control group (Ctr) received equal volumes of PBS at identical intervals. Animals of the SFN + MPTP and EtOH + MPTP groups received one injection of 10 mg/kg of body weight SFN or 14.1% EtOH (the solvent for SFN), respectively, 12 h prior to the MPTP treatment. Animals were sacrificed 90 min following the last MPTP injection, and total Neat1 and Neat1_2 levels were determined by quantitative RT-PCR analysis according to the protocol described in the Methods section. Our analysis did not reveal up-regulated Neat1 levels in striatum or brainstem samples within the treated study groups (Fig. 2a,c). In contrast, Neat1_2 was significantly up-regulated as compared to the control (Ctr) in each group treated with MPTP, most prominently in the MPTP + SFN co-treated animals, in both investigated brain areas (Fig. 2b,d). Thus, we concluded that while a strong effect of SFN on *Neat1* expression *in vivo* was not detectable under our conditions, a weak co-inducing effect with MPTP might exist. However, comparisons between MPTP, SFN + MPTP, and EtOH + MPTP treated samples indicated that EtOH, the solvent of SFN, might also contribute to Neat1 level modulation. As this can interfere with the interpretation of data regarding SFN effect, we limited our further comparisons to Ctr and MPTP only samples. Nonetheless, we note a significant difference between Neat1_2 levels in the striatum of MPTP-treated and MPTP + SFN co-treated animals (Fig. 2d).

### The alterations in Neat1 levels differ in early and later phases of responses to toxin treatment in MPTP PD models

The observation of changes in total Neat1 and Neat1_2 levels upon MPTP treatment made us curious to determine whether the two isoforms of Neat1 show differential expression in early and progressed stages of MPTP-induced neurodegeneration. For this we used treatments which can be considered as presymptomatic (PSS) and early symptomatic (ESS) stages of MPTP toxin induced PD models^19,20^. Accordingly, we compared changes in the expression of Neat1 and Neat1_2 in brain samples of MPTP-treated mice 90 min and 7 days following MPTP toxin treatment (in the following we refer to the two treatment regimens as the models of presymptomatic (PSS) and early symptomatic (ESS) stages, respectively). The levels of Neat1 and Neat1_2 were assessed by quantitative PCR as described in the Methods section in four brain regions: brainstem, striatum, cortex, and cerebellum. For clarity, shifts in Neat1:Neat1_2 proportions were detected within specific investigated brain areas by determining the relative amounts of the total and long isoform of the lncRNA, as described above.

**Fig. 3:**
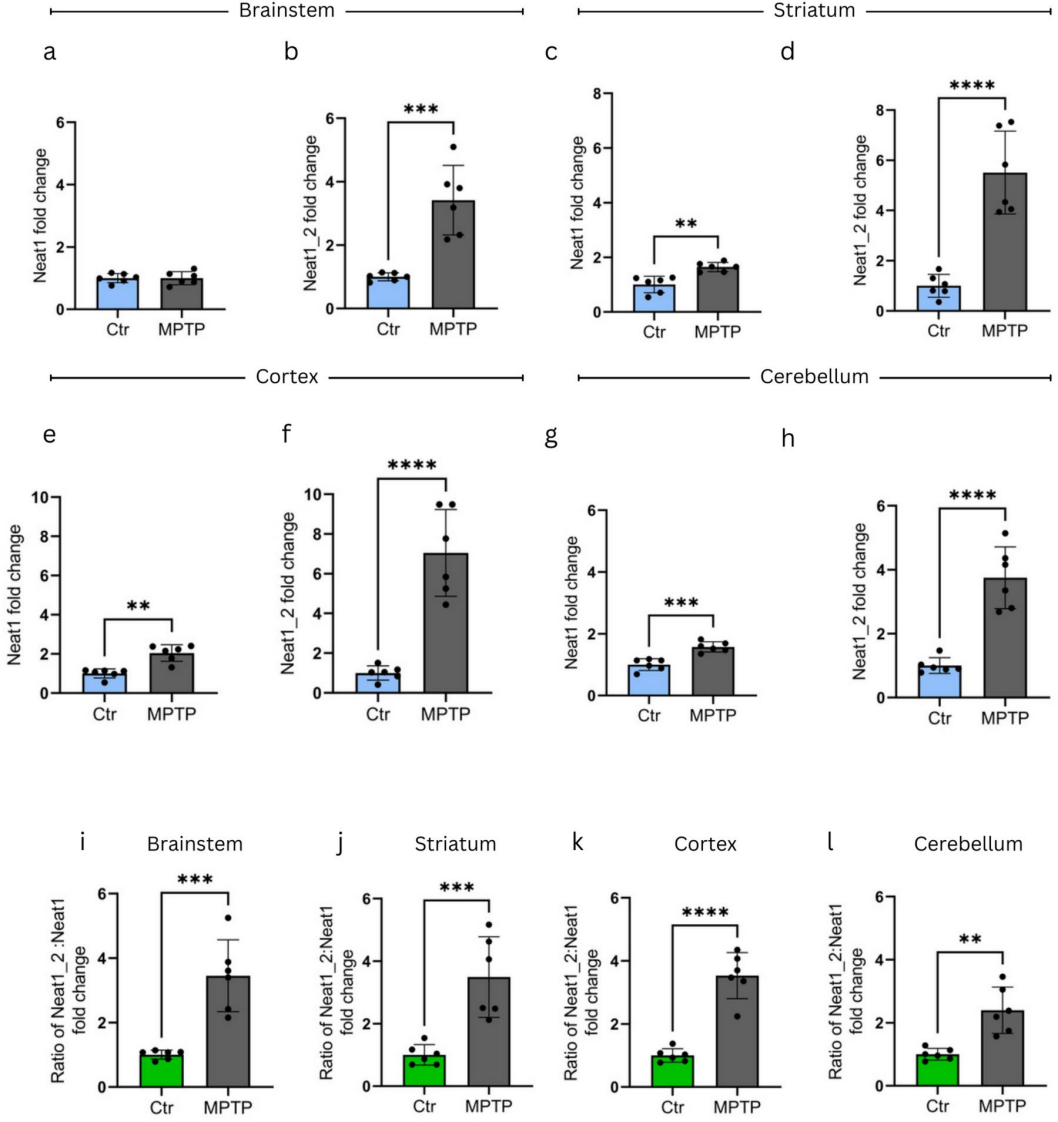
Changes in Neat1 expression* in vivo *in the PSS MPTP model. Neat1_2 level is significantly increased 90 min following MPTP treatment as compared to control animals in (**b**) brainstem (fold change = 3.4, p = 0.0003), (**d**) striatum (fold change = 5.5, p < 0.0001), (**f**) cortex (fold change = 7.1, p < 0.0001), and (**h**) cerebellum (fold change: 3.8, p < 0.0001). Total Neat1 level shows no up-regulation in the brainstem (**a**) while it is slightly, but significantly increased in (**c**) striatum (fold change = 1.6, p = 0.0011), (**e**) cortex (fold change = 2, p = 0.0022) and (**g**) cerebellum (fold change = 1.6, p = 0.0002) samples upon MPTP treatment. Neat1_2:Neat1 ratio in MPTP treated animals compared to control mice in (**i**) brainstem (fold change ratio = 3.5, p = 0.0003), (**j**) striatum (fold change ratio = 3.5, p = 0.0010), (**k**) cortex (fold change ratio = 3.5, p < 0.0001), and (**l**) cerebellum (fold change ratio = 2.4, p = 0.0011). For the assessment of data distribution the Shapiro–Wilk test was used. Except for graph (**e**), all datasets showed normal distribution, therefore unpaired t-test was implemented. In the case of graph (**e**), Mann-Whitney test was performed (n = 6). Values are plotted as mean ± SD. Datapoints represent values detected in individual animals.* Ctr* control, *MPTP* 1-methyl-4-phenyl-1,2,3,6-tetrahydropyridine* ,NEAT1* Nuclear enriched abundant transcript 1, *SFN* sulforaphane, *MPP* + *1*-methyl-4-phenylpyridinium.

In the PSS model, MPTP treatment resulted in significant increase in Neat1_2 levels (Fig. 3b,d,f,h), showing the most prominent up-regulation in striatum (Fig. 3d) and cortex (Fig. 3f) samples. The detected increases (4–5-fold as compared to Ctr) are comparable to the range of changes detected in previous experiments in corresponding samples (Fig. 2b,d, approximately 4-fold increases). While the levels of Neat1 were also significantly increased in three of the investigated brain areas, the increase was not as pronounced as in the case of the long isoform (Fig. 3a,c,e,g). A comparison of Neat1 Ct values with that of internal controls of Q-PCR reactions clearly indicated that the significant increases in Neat1_2:Neat1 ratios in MPTP treated animals compared to control mice (Fig. 3i,j, k, and l) were primarily due to the prominent increase of the long isoform of the lncRNA. We note here that the increases in Neat1_2 level were detected in comparison with total Neat1 levels, which differ significantly in different brain regions.

**Fig. 4:**
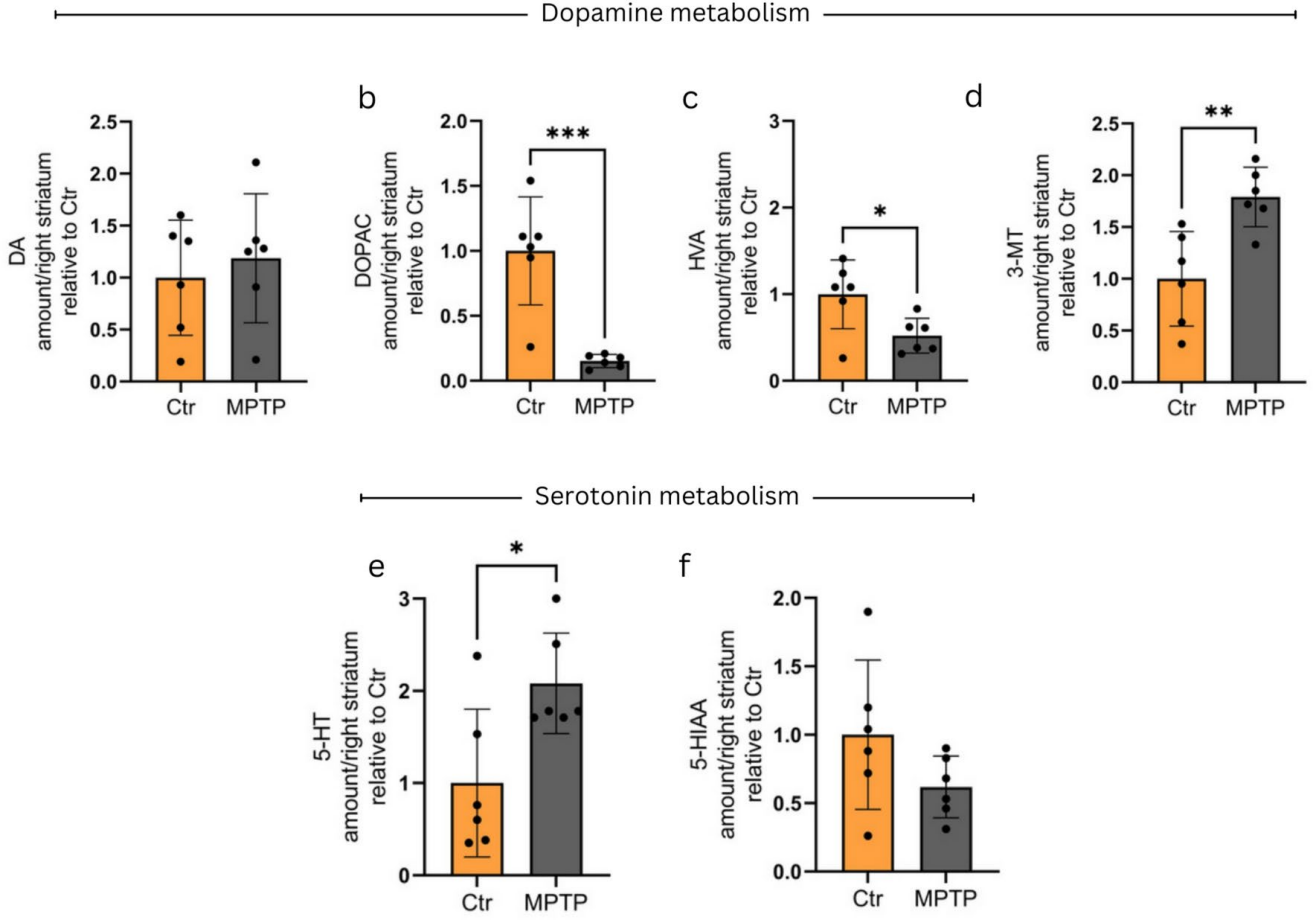
Assessment of DA, 5-HT, and metabolite levels in the PSS MPTP model. No significant change is observable in the striatal levels of DA (**a**) 90 min following MPTP treatment, whereas DOPAC (**b**) and HVA (**c**) levels are significantly decreased, and 3-MT (**d**) levels are significantly increased in the toxin-treated group compared to control animals (DOPAC: p = 0.0006; HVA: p = 0.0252; 3-MT: p = 0.0050). MPTP treatment results in an increase in the levels of 5-HT (**e**) (p = 0.0260), while in the case of the 5-HT metabolite, 5-HIAA (**f**), a tendency towards decreased levels is observed upon toxin treatment. The amounts of indicated metabolites are given in arbitrary units expressed as relative value compared to control average. For the assessment of data distribution the Shapiro–Wilk test was used. Except for graph (**e**), all datasets showed normal distribution, therefore unpaired t-test was implemented. In the case of graph (**e**), Mann-Whitney test was performed (n = 6). Values are plotted as mean ± SD. Datapoints represent values detected in individual animals. Abbreviations: DA: dopamine; DOPAC: 3,4-Dihydroxyphenylacetic acid; HVA: Homovanillic acid; 3-MT: 3-Methoxytyramine; 5-HT: serotonin; 5-HIAA: 5-Hydroxyindoleacetic acid.* Ctr* control, *MPTP* 1-methyl-4-phenyl-1,2,3,6-tetrahydropyridine* ,NEAT1* Nuclear enriched abundant transcript 1, *SFN* sulforaphane, *MPP* + *1*-methyl-4-phenylpyridinium.

Next, we asked if there is correlation between the changes in Neat1_2 level and alterations in the levels of dopamine (DA) and serotonin (5-HT) metabolites. In the PSS model the levels of DA did not decrease significantly in the toxin-receiving groups (Fig. 4a). On the other hand, MPTP treatment significantly reduced the levels of the two other DA metabolites, 3,4-Dihydroxyphenylacetic acid (DOPAC) and homovanillic acid (HVA) (Fig. 4b,c) and increased the level of 3-Methoxytyramine (3-MT) (Fig. 4d). Regarding 5-HT and its metabolites, MPTP treatment resulted in increase in the levels of the neurotransmitter (Fig. 4e), while the level of 5-Hydroxyindoleacetic acid (5-HIAA) showed a tendency to decrease upon toxin treatment (Fig. 4f). Analyses of correlations between levels of Neat1_2 and those of the investigated metabolites revealed a significant negative correlation between DOPAC levels and the long isoform (Supplementary Fig. 3. b), and positive correlation in relation with 3-MT and 5-HT (Supplementary Fig. 3. d and e). No correlation was observed with the other assessed metabolites (Supplementary Fig. 3. a, c, and f).

**Fig. 5:**
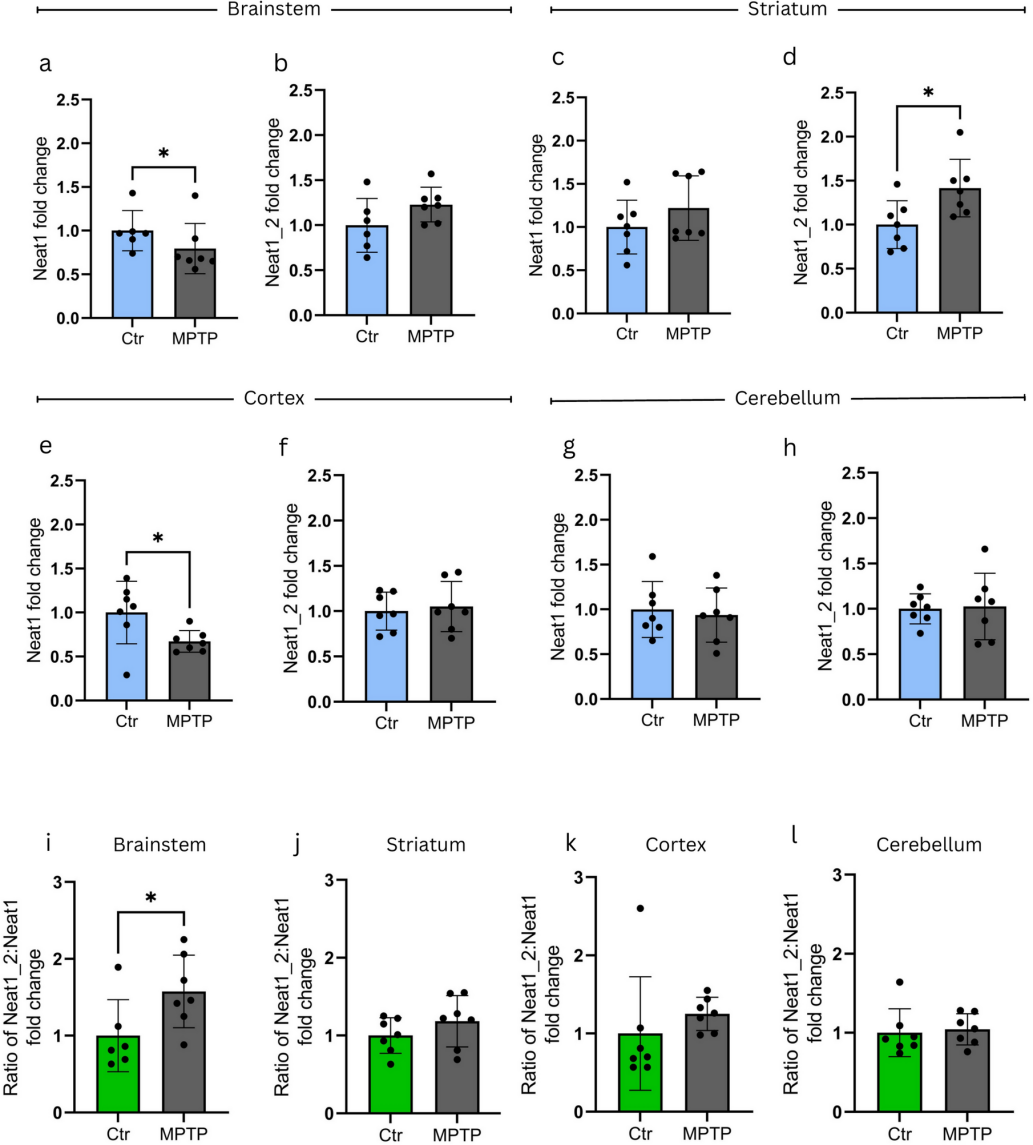
Changes in Neat1 expression *in vivo* in the ESS MPTP model. Seven days following MPTP treatment, Neat1 level shows significant decrease in (**a**) brainstem (fold change = 0.8; p = 0.0466) and in (**e**) cortex (fold change = 0.7; p = 0.0392) samples of MPTP-treated animals. No changes in the expression of Neat1_2 are observable in either brainstem (**b**) or cortex (**f**). No differences are observable in the levels of either Neat1 (**g**) or Neat1_2 (**h**) between study groups in the cerebellum, and only a slight, though significant increase in Neat1_2 level is seen in the striatum of the MPTP toxin-treated group (**d**) (fold change = 1.4, p = 0.0236), while in the total NEAT1 levels there is no significant difference (**c**). Neat1_2:Neat1 ratio reveals significant increase in Neat1_2 level only in brainstem samples of MPTP-treated animals (**i**), while no significant difference was observable in this respect between the two study groups in any other investigated brain regions (**j**, **k**, and **l**). For the assessment of data distribution the Shapiro–Wilk test was used. In case of normal distribution, unpaired t test was implemented (**b**, and **d**-**j**), otherwise the non-parametric Mann–Whitney test was performed (**a**, **c**, **k**, and **l**) (n = 6–7). Values are plotted as mean ± SD. Datapoints represent values detected in individual animals. *Ctr* control, *MPTP* 1-methyl-4-phenyl-1,2,3,6-tetrahydropyridine*, NEAT1* Nuclear enriched abundant transcript 1, *SFN* sulforaphane, *MPP* + *1*-methyl-4-phenylpyridinium

To model an extended toxin effect (ESS model), animals received MPTP injections 5 times, with 2 h between injections, and were sacrificed 7 days following the last toxin administration. Controls received PBS injections following an identical regime. In this model, a significant decrease in the level of Neat1 was observable in brainstem and in cortex samples of MPTP-treated animals (Fig. 5a,e); this, however, was not accompanied by a change in Neat1_2 level as compared to control (Fig. 5b,f). In the other investigated brain areas, except for a slight, but significant increase in the striatal Neat1_2 levels of the MPTP treated animals, no differences were observed in the levels of either Neat1_2 or Neat1 between the study groups (Fig. 5c-d,g-h). The ratio of Neat1_2 to Neat1 levels indicates a significant increase only in brainstem samples of MPTP-treated animals (Fig. 5i), while no significant difference was observed in this respect in any other investigated brain regions between the two study groups (Fig. 5j,k, and l).

**Fig. 6:**
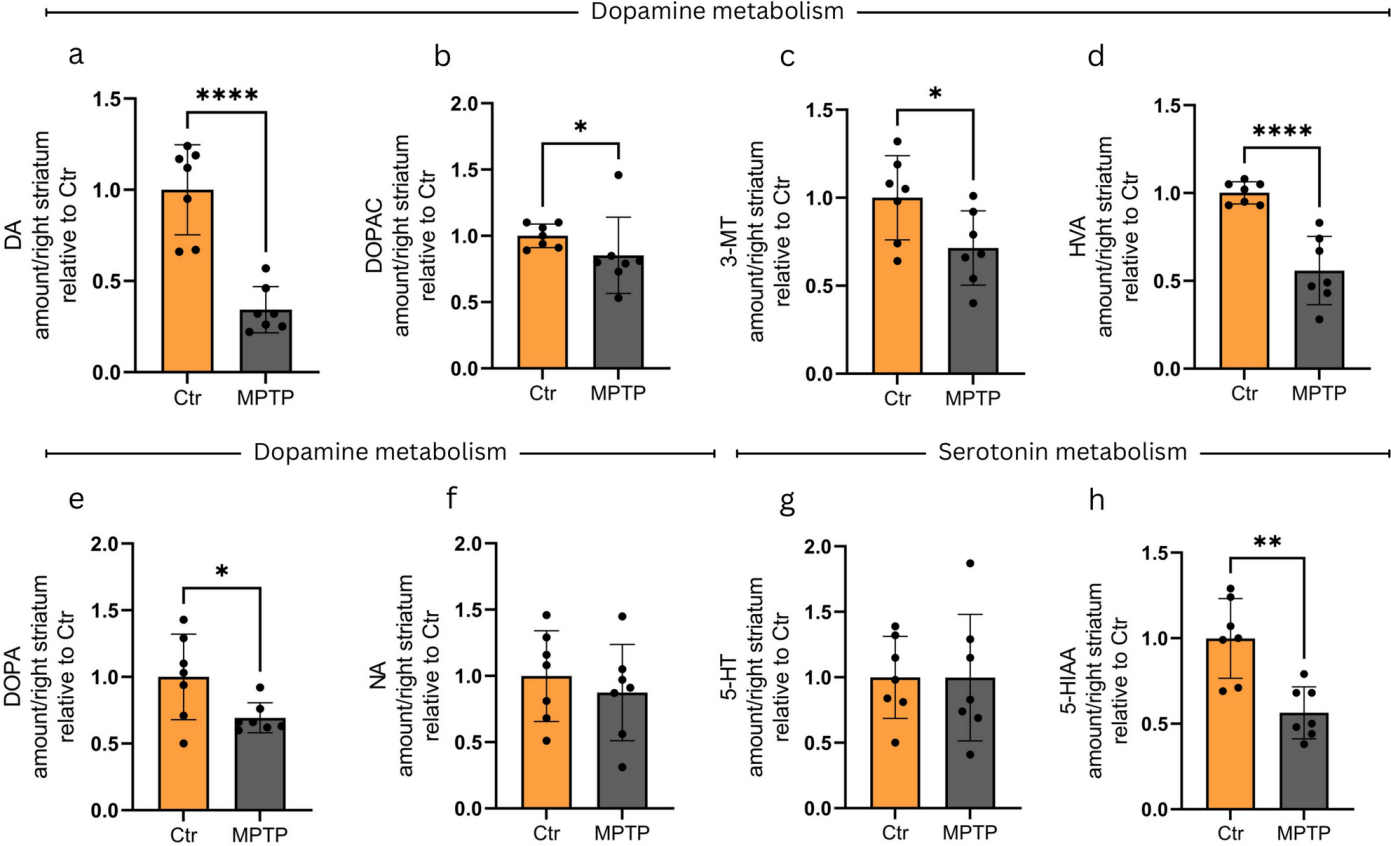
Assessment of DA, 5-HT, and metabolite levels in the ESS MPTP model. Significant decreases are observable in toxin treated animals compared to control mice in the striatal levels of (**a**) DA (p < 0.0001), (**b**) DOPAC (p = 0.0251), (**c**) 3-MT (p = 0.0358), (**d**) HVA (p < 0.0001) (**e**) DOPA (p = 0.0490), and (**h**) 5-HIAA (p = 0.0014) seven days following MPTP treatment. The levels of (**f**) NA, and (**g**) 5-HT are not altered. The amounts of indicated metabolites are given in arbitrary units expressed as relative value compared to control average. For the assessment of data distribution the Shapiro–Wilk test was used. For graphs (**b**) and (**e**) the non-parametric Mann–Whitney test was performed. In case of all other analyses presented in the figure unpaired t-test was implemented (n = 7). Values are plotted as mean ± SD. Datapoints represent values detected in individual animals. *Ctr* control, *MPTP* 1-methyl-4-phenyl-1,2,3,6-tetrahydropyridine* ,NEAT1* Nuclear enriched abundant transcript 1, *SFN* sulforaphane, *MPP* + *1*-methyl-4-phenylpyridinium.

Analyses of samples of MPTP-treated animals in the ESS model revealed a significant decrease in striatal DA (Fig. 6a), DOPAC (Fig. 6b) 3-MT (Fig. 6c), HVA (Fig. 6d), DOPA (Fig. 6e), and 5-HIAA (Fig. 6h) levels in the toxin-treated group, whereas the levels of NA and 5-HT were not altered (Fig. 6f,g). Statistical analysis, however, revealed no significant correlation between the levels of any of the investigated metabolites and that of the lncRNA (Supplementary Fig. 4.).

## Discussion

Our data presented here show that in SH-SY5Y cells NEAT1 up-regulation can be achieved by both MPP + and SFN treatment. However, while MPP + treatment results in decreased cell viability, SFN treatment does not decrease, but rather enhances the viability of the cells, despite the similar effects of the two compounds on NEAT1 expression. Moreover, SFN decreases the rate of cell apoptosis caused by MPP + . The observation that the two compounds both resulted in NEAT1 up-regulation, yet exerted opposing effects on the viability of cells prompted us to examine (a) if the opposite effects of NEAT1 up-regulation via SFN and MPP + can be recapitulated *in vivo,* (b) whether there is a difference in the expression of the two isoforms of the lncRNA and (c) if yes, how do the levels of Neat1 variants change in PSS and ESS MPTP models of PD. Our results with a small number of animals (3 in each group) gave a negative answer to the first question, as under the conditions we used SFN did not lead to strong Neat1 up-regulation throughout the brain regions of interest (Supplementary Fig. 2.). On the other hand, the results demonstrated that *in vivo* low-dose MPTP administered alone or in combination with SFN led to a prominent increase in the levels of the long isoform of the lncRNA within a short time window following toxin treatment (Fig. 2). We observed also that the solvent of SFN, EtOH affected Neat1 level when used in combination with MPTP. Therefore, we abandoned the analysis of *in vivo *SFN effects. Consequently, we report here results of analyses done to explore the effect of MPTP on the levels of total and long isoform of Neat1 in different time windows following drug administration. Our RNA level determination was based on quantitative RT-PCR assays extended to four well determined brain regions, and we only compared RNA levels within a particular brain area. (While we report here only on comparisons between MPTP treated and control groups each consisting of 6 animals, we note that similar comparisons between groups treated with MPTP + SFN, MPTP + EtOH and appropriate controls (as shown in Fig. 2.) gave very similar results.)

For the analyses of rapid and delayed responses to toxin we used established presymptomatic (PSS) and early symptomatic (ESS) MPTP-induced PD models.

In the PSS model, no alterations of DA and 5-HT levels were detected 90 min after toxin administration, indicating no permanent loss of dopaminergic neurons. On the other hand, in this model an elevated level of the long Neat1 isoform was detectable in each investigated brain area as compared to mock (PBS)-treated controls. The increase of Neat1_2 level resulted in a 2.5–3-fold change in the Neat1_2:Neat1-total ratio. Interestingly, a similar grade of shift in the ratio of Neat1 long isoform and total Neat1 level was detected in each of the brain regions studied, though we estimated an up to 5-fold difference in basal Neat1 levels among the studied brain regions (data not shown). Further studies are required to elucidate whether this change is a result of transcription or RNA maturation changes in specific cell types, and whether a change in the half-life of either of the two Neat1 isoforms plays a role.

In contrast to what was found in the PSS, in the ESS model the significant 2.5–3-fold increase in Neat1_2 level as compared to Neat1-total level in MPTP treated animals was not detected. The sharp change in the ratio of the two Neat1 isoforms is diminished by 7 days following MPTP treatment. In this model we detected a smaller, though significant increase in Neat1_2:Neat1 ratio remaining only in the brainstem of MPTP treated animals. By that time severe alterations in the levels of DA metabolites are observable, indicating a more severe effect on dopaminergic neurons.

Overall, these findings suggest that a shift between the two isoforms of the Neat1 lncRNA accompanies the early response to central nervous system (CNS) damage. Whether this is part of an active protective measure or a secondary response to other molecular alterations caused by the toxin needs further elucidation. Nonetheless, an increasing body of evidence supports the former notion. Accordingly, NEAT1_2, but not NEAT1_1, was shown to be up-regulated in spinal motoneurons in the early stages of ALS^3,11,14,21^. According to Nishimoto *et al.* at the early stage of the disease, cellular stress promotes NEAT1_2 up-regulation: however, continuation of the stressful stimulus leads to an increased expression of NEAT1_1. The loss of cells results from the lack of protective processes due to the lack of NEAT1_2 and paraspeckles, while aggravated toxicity is caused by an elevated level of NEAT1_2.

A protective role for the longer isoform of NEAT1 has been proposed in other neurodegenerative diseases as well. Neat1_2 was found to be up-regulated in various models of polyglutamine (polyQ) repeat diseases such as spinocerebellar ataxia types 1, 2, 7, and HD. In the latter, Neat1_2 was found to protect cells from toxicity induced by mutant huntingtin (mHTT)^22^. Our observations with the use of PSS and ESS models seem to be in accord with these observations, suggesting that an increased level of the longer isoform is associated with a protective early response.

In respect of PD, a number of reports argue for the detrimental role of NEAT1 based on studies utilizing NEAT1 down-regulation in SH-SY5Y, SK-N-SH, and SK-N-AS cells and in *in vivo* models of the disease^23–34^. In line with our results, these studies show a dose-dependent increase of NEAT1 levels upon MPP + treatment *in vitro*^29^. However, they report that siRNA knockdown of NEAT1 in MPP + treated cells increases cell viability and decreases apoptosis *in vitro*^24–35^, and suppresses autophagy and increases the number of TH + neurons *in vivo* in MPTP- treated mice^23,29,32,33^. However, when reporting gene expression changes, none of these studies distinguish between the long and short isoforms of NEAT1.

In these studies, siRNA treatment was generally introduced prior to the application of the toxin treatment in both *in vitro*^27,28^ and *in vivo*^29,31,33^ experiments. Furthermore, a higher dose of MPTP was typically used to establish the mouse PD model, with doses of 20–30 mg/kg applied one to four times a day for up to three consecutive weeks^23,24,29,31,33,35^. These *in vivo* models resemble the ESS model presented in our study, in which the up-regulation of Neat1_2 was no longer observed. One might speculate that under these conditions, such as when siRNA treatment is applied prior to the start of the toxin treatment, a beneficial effect might be due to the down-regulation of the short Neat1 isoform, which, as discussed above, would otherwise lead to aggravated toxicity^11^. It would be interesting to see the result of targeted down-regulation of the long isoform.

Findings reported by Simchovitz *et al*. support the notion of the protective role of NEAT1_2 in PD models^16^. Based on their observed robust NEAT1 – particularly NEAT1_2 – up-regulation in various models of the disease, they propose that increased NEAT1 level is due to up-regulation of the paraspeckle associated long isoform, and consider the enhanced paraspeckle formation as a protective measure against neurotoxicity. Their analysis of *post mortem* substantia nigra samples of PD patients however, showed higher increase for the long isoform. This finding seemingly contradicts our hypothesis on the shift of the amount of long- towards the short isofom upon disease progression, as we observed it in an animal model of PD. At present we do not have enough data to resolve this incongruence which might result from the significant differences in disease stages and also from a wide range of factors influencing NEAT1 levels in human, including hormonal changes^36^, comorbidities^37,38^, and medications^16^, which make direct reconciling of results obtained from disease models and human samples often challenging.

In our *in vitro* experiments, we used SFN for pharmacological up-regulation of NEAT1. Our data clearly prove that an increase of NEAT1 level by SFN causes no harm in the *in vitro* neuroblastoma cell PD model. Although this seems to contradict the data from the siRNA experiments mentioned above, this contradiction could result from the different time frames of the experiments and the non-selective inhibition of the distinct NEAT1 isoforms, as detailed previously.

The up-regulation of Neat1_2 observed in our PSS MPTP mouse model could be explained as part of an early protective mechanism of the cells upon MPTP -induced oxidative damage. While the exact mode by which MPTP exerts neurotoxicity is not yet fully elucidated, one of the most well-known mechanisms is *via* inhibition of the mitochondrial electron transport chain complex I^39^, which may support this suggestion. Considering that Neat1 plays an important role in protective functions against oxidative stress, up-regulation of Neat1_2 could be part of the activation of a protective mechanism.

**Fig. 7:**
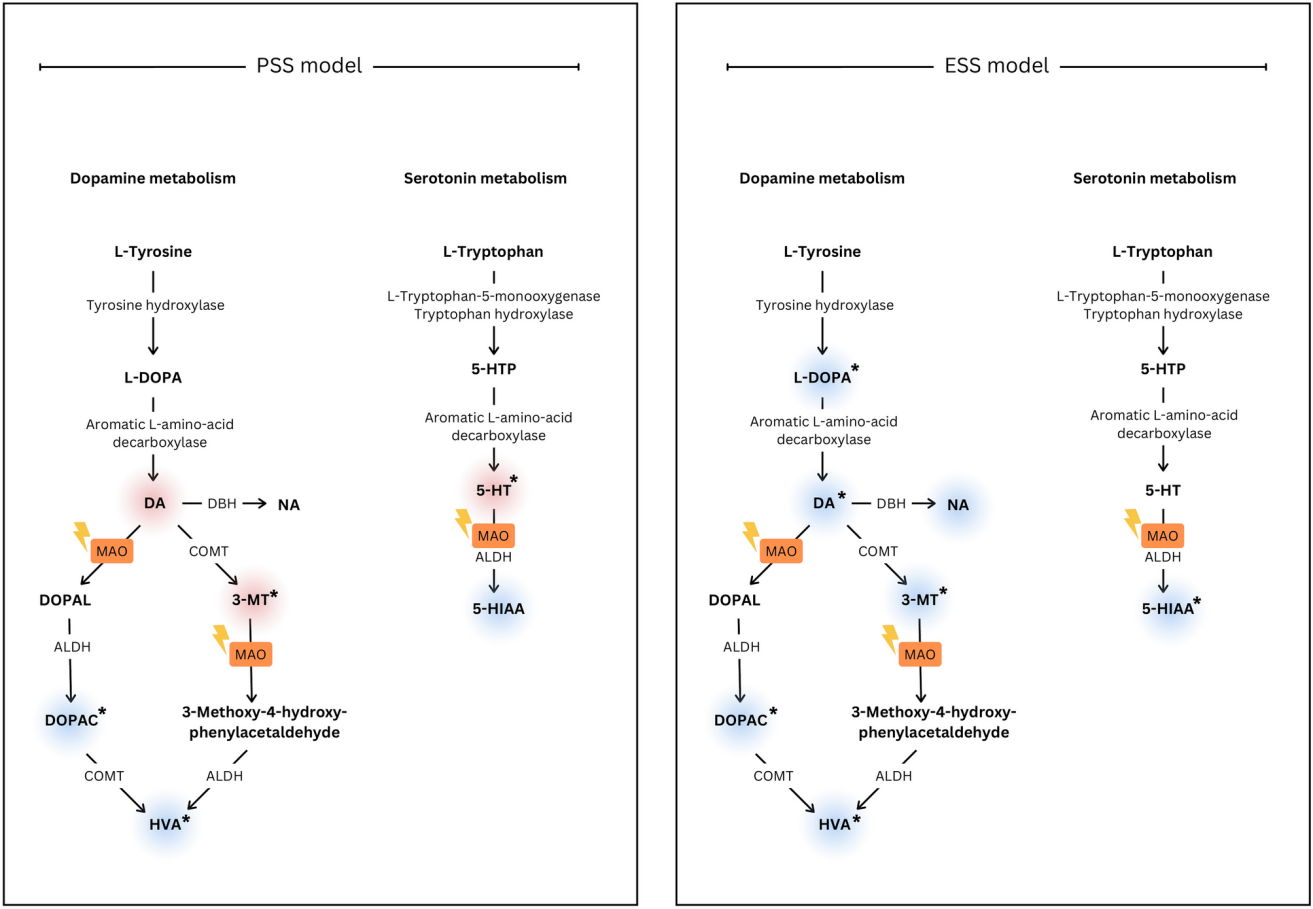
Overview of dopamine and serotonin metabolism with a focus on the PSS and ESS MPTP models used in this study. Analysis of DA and 5-HT metabolites in striatum samples of animals following short term/low dose MPTP treatment (PSS MPTP model, left panel) revealed increase in the levels of MAO enzyme substrates such as DA, 3-MT, and 5-HT, whereas the levels of compounds downstream to the enzyme, such as DOPAC, HVA, and 5-HIAA were decreased. The alterations are in accord with changes expectable upon MAO inhibition: upstream products are accumulated whereas downstream metabolites are present in decreased levels. On the contrary, in the ESS MPTP model (right panel) all the investigated metabolites show significant decrease, or a tendency of decreased levels upon toxin treatment. Substrates showing an increase in their levels are marked with a red background, whereas those with decreased levels are indicated in blue. The asterisk (*) indicates significant difference when comparing the MPTP-treated group to the control cohort.* Ctr* control, *MPTP* 1-methyl-4-phenyl-1,2,3,6-tetrahydropyridine* ,NEAT1* Nuclear enriched abundant transcript 1, *SFN* sulforaphane, *MPP* + *1*-methyl-4-phenylpyridinium.

An interesting aspect of our study is the change observed in DA and 5-HT metabolite levels in striatum samples of animals following short-term/low-dose MPTP treatment. We revealed an increase in the levels of monoamine oxidase (MAO) enzyme substrates such as DA, 3-MT and 5-HT, and a decrease in the levels of DOPAC, HVA and 5-HIAA. These alterations correspond to changes that can be expected upon MAO inhibition, where upstream products accumulate as their metabolism is impaired, whereas diminishing amounts of downstream metabolites are produced (Fig. 7).

The two monoamine oxidases MAO-A and B both play roles in DA and 5-HT metabolism ^40,41^. When MPTP enters the brain, it is converted to 1-methyl-4-phenyl-2,3-dihydropyridinium (MPDP +) by MAO-B enzymes in glial cells and serotoninergic neurons^42,43^. Alongside MAO-B, MAO-A also plays a role in MPTP conversion and contributes to MPTP toxicity^44^.

The alterations we observed in DA and 5-HT metabolite levels upon low-dose/short-term MPTP treatment (PSS model) thus could be explained to be due – at least partially – to MPTP blocking MAO enzyme activity either as a competing substrate or by other mechanisms. The exploration of pathways through which the changes of metabolite levels are linked to the changes in the levels of Neat1 isoforms – if any do exist – requires further studies.

In conclusion, based on our results we argue for a double role of NEAT1 which can accommodate the contradictory findings observed in different PD models. According to our hypothesis, upon MPTP stress a protective role of Neat1_2 is initially dominant. With progression of the pathology the level of Neat1_2 decreases, resulting in a shift in the ratio of the two isoforms, accompanied by CNS damage. Deciphering the exact changes in levels and roles of the isoform variants throughout the course of neurodegeneration holds the potential of providing further insight into the mechanisms underlying neurological disorders such as PD.

## Methods

### *In vitro* SH-SY5Y cell model

For an *in vitro* model of PD, the SH-SY5Y human neuroblastoma cell line was used (cells were kindly made available by the laboratory of Professor László Vigh, Biological Research Centre (BRC), Szeged, Hungary). Cells were cultured at 37 °C at 5% CO2 in Dulbecco’s Modified Eagle Medium/Nutrient Mixture F-12 (DMEM-F12; Lonza, Basel, Switzerland) supplemented with 10% foetal bovine serum (FBS; Gibco, Thermo Scientific, Waltham, MA, USA), 2 mM L-glutamine, and antibiotics penicillin and streptomycin (1%-1% each). Cell cultures were trypsinised and passaged on every third day. The same incubation conditions were implemented during the treatment periods. For treatments cells were seeded twenty-four hours prior to treatment at a density of 2.2*10^6^ cells on 10 cm (10 ml) petri dishes for RNA and FACS analysis. For viability assays cells were distributed into 96 well plates (5*10^4^ cells/100 μl/well). For cell treatments, MPP + (cat.no.: D048, Sigma-Aldrich, Darmstadt, Germany) was dissolved in phosphate-buffered saline (PBS), while SFN (cat.no.:574215, Sigma-Aldrich, Darmstadt, Germany) was dissolved in ethanol.

#### Determination of cell viability

Cell viability measurements were carried out with Cell Counting Kit-8 (CCK-8) according to the instructions of the manufacturer (Sigma-Aldrich, St. Louis, Missouri, USA). In brief, cells in 96 well plates were treated with different reagents to induce neurodegeneration and/or neuroprotection as described in the text. Following the treatment, culture medium was carefully aspirated and was substituted with a mixture of fresh medium, and CCK-8 assay was performed. Cells were incubated for two hours at 37 °C. For absorbance measurement a Gen5™ Microplate Reader (BioTek Instruments, Inc., Winooski, VT, USA) was used. Absorbance was measured at 450 nm and 650 nm to exclude the differences originating from background absorbance. Changes in cell viability were calculated with the use of the difference in absorbance at 650 nm and 450 nm.

#### Analysis of apoptosis by fluorescence-activated cell sorting flow cytometry

For apoptosis analysis Annexin V-FITC Apoptosis Detection Kit was used (eBioscience™, Thermo Fisher Scientific Inc., Waltham, Massachusetts, USA). Following treatments as described in the text, both adherent and floating cells were collected, washed with PBS and resuspended in 300 μl 10X Binding Buffer. 5 μl Annexin V-FITC was added to each sample and incubated for 15 min at room temperature. Following this, 5 µl propidium iodide (PI) solution (8 ng/μl) was added and the samples were kept on ice. Cells were analyzed by fluorescence-activated cell sorting (FACS) flow cytometry on a BD FACS Calibur flow cytometer. Data was analyzed by the CellQuest pro software.

### *In vivo* mouse model

For the experiments presented in this study 10–12 weeks old male C57BL/6 J mice were utilized. The strain was originally obtained from Jackson Labs (Jackson Laboratories, Bar Harbor, ME, USA) and further bred in our institutional animal facility. The animals were kept under standard laboratory conditions (12–12 h light–dark cycle, free access to food and water). All animal experiments were carried out in accordance with the European Communities Council Directive (86/609/EEC) and approved by the local animal care committee (number of ethical approval: XI./2692/2020). The authors complied with the ARRIVE guidelines.

For SFN treatment, SFN was dissolved in absolute EtOH to prepare a stock solution of 40 mM, which was further diluted with 0.9% NaCl solution to the desired concentration depending on the treatment, and administered via i.p. injection at a maximum volume of 10 µl/gram of body weight. For controls, the animals received 14.1% EtOH solution prepared in 0.9% NaCl solution (EtOH concentration corresponding to the EtOH content of the solution for the highest SFN dose implemented).

MPTP (cat.no.: HY-15608, MedChemExpress, NJ, USA) was dissolved in PBS and administered via i.p. injection. For the PSS model animals received 3 injections of 15 mg/kg body weight MPTP solution, with 2 h between injections, and were sacrificed 90 min following the last treatment. For the ESS model mice were treated with a total of 5 injections with 2 h between injections, with the same dose/treatment as described above. Controls to MPTP treatments received injections of PBS following the regime of drug treatments. Animals of the ESS model were terminated 7 days following MPTP treatment. For this, the mice were deeply anesthetized with isoflurane (Forane; Abott Laboratories Hungary Ltd., Budapest, Hungary), followed by thoracotomy and transcardial perfusion with artificial cerebrospinal fluid for 2 min at 10 rpm by an automatic peristaltic pump. After termination brains were rapidly removed on ice and cut into half in a sagittal section. For gene expression and HPLC analysis four brain regions (brainstem, cerebellum, striatum, and cortex) were separated from both right and left hemispheres, and the samples were stored at -80 °C until further use. The experiments involving gene expression analysis were all carried out using the left-side samples, high-performance liquid chromatography (HPLC) measurements were done with the use of the right-side samples.

### RNA sample preparation and gene expression analysis

For RNA analysis, cells on 10 cm plates were lysed in 750 μl TRI Reagent (Sigma-Aldrich, St. Louis, Missouri, USA), scraped and collected into Eppendorf tubes. Subsequent RNA isolation was done following the instructions of the manufacturer (Sigma-Aldrich, St. Louis, Missouri, USA).

For RNA extraction from animal samples, frozen mouse brain tissue was homogenized in TRI reagent (Molecular Research Center Inc., Cincinatti, OH, USA) with an ultrasound homogenizer (UP100H, Hielscher Ultrasound Technology, Germany; amplitude: 100%, cycle: 0.5). Total RNA was isolated following the instructions of the TRI reagent manufacturer (Molecular Research Center Inc., Cincinatti, OH, USA). RNA concentration was determined using MaestroNano micro-volume spectrophotometer (MaestroGenInc, Hsinchu, Taiwan). The quality of RNA samples was tested by determining absorbance ratio at 260:280, and samples were stored at -80 °C until further analysis.

To detect lncRNA levels, 1 μg mouse-tissue derived and 2 μg cultured cell total RNA was used for cDNA synthesis. In order to remove genomic DNA prior to cDNA synthesis, RNA was treated with RNase-free DNase I following the instructions of the manufacturer (DNase I, RNase free, Thermo Fisher Scientific Inc., Marietta, OH, USA). cDNA was reverse-transcribed with random hexamer primer with the use of the RevertAid First Strand cDNA Synthesis Kit according to the manufacturer’s instructions (Thermo Fisher Scientific Inc., Marietta, OH, USA). qPCR reactions were carried out with SYBER green detection (RT2 SYBR Green Mastermix (qPCRBIO)) in a final volume of 20 μl. 18S rRNA was used as a housekeeping gene. In the case of animal experiments commercially available 18S rRNA primers were purchased from Applied Biosystems (Carlsbad, CA, USA) and for PCR reactions TaqMan probe mix (qPCRBIO) was used. All real time PCR reactions were carried out in a CFX96 thermocycler (Bio-Rad). Primer sequences and cycling conditions are listed in Table 1 and 2 respectively.

### Determination of biogenic amines and related compounds by HPLC

PSS model: The levels of biogenic amines and related compounds of the halved striatal samples were analysed with a previously developed HPLC-ECD method ^45^. Briefly, the following materials can be determined electrochemically: levodopa (L-DOPA), 3,4-dihydroxyphenylacetic acid (DOPAC), norepinephrine (NA), 5-hydroxyindoleacetic acid (5-HIAA), homovanillic acid (HVA), dopamine (DA), 5-hydroxytryptamine (5-HT), 3-methoxytyramine (3-MT), and their internal standards: 3,4-dihydroxybenzylamine (DHBA, for L-DOPA, DOPAC, NA, and 5-HIAA), isoproterenol (IPR, for HVA, and DA), and 5-hydroxy-N-ω-methyltryptamine (NM-5HT, for 5-HT, and 3-MT). As a result of a failed limit of detection levels of L-DOPA and NA in most samples, only the remaining monoamine levels are provided in the Results section.

ESS model: The catechol- and indole-amine content of extracts from the striatum of mice was determined by HPLC. The extract of the brain area samples was prepared by ultra-sonication with 0.01 M PCA solution, which contained the following components: theophylline (as an internal standard) at a concentration of 10 µM, and 0.5 mM sodium metabisulfite (antioxidant for biogenic amines). The tissue extract was centrifuged at 3510 g for 10 min at 4 °C and the pellet was saved for protein measurement ^46^. The perchlorate anion was precipitated from the supernatant with 4 M dipotassium phosphate and removed by a subsequent centrifugation. The samples were used immediately or kept at -20 °C until analysis. Quantification of the analytes was performed using an online sample enrichment with column switching technique and liquid chromatographic separation. Solid phase extraction (SPE) was performed on a HALO Phenyl-Hexyl (75 × 2.1 mm diameter, 5 µm) column with buffer "A" for the appropriate time. And the separation was completed in connection with an ACE Ultra Core Super C-18 (150 × 2.1 mm diameter, 5 µm) analytical column. The following mobile phases were used for the separation [“A” 10 mM potassium phosphate, 0.25 mM EDTA and “B” 0.45 mM octane sulfonyl acid sodium salt, 8% acetonitrile (v/v), 2% methanol (v/v), pH 5.2] with a flow rate of 250 µl/min, with a step gradient ^47^. A Shimadzu LC-20 AD HPLC system was used for the analysis. Series-connected amperometric (BAS CC-4) and UV (Agilent 1100 variable wavelength) detectors were used for the signalling of the analytes. The signal of the monoamines was recorded electrochemically using an oxidation potential of 0.73 V, while the internal standard was recorded by its UV absorption at 253 nm. Concentrations were calculated by a two-point calibration curve internal standard method: (Ai * f * B)/(C * Di * E) (Ai: Area of biogenic amine component; B: Sample volume; C: Injection volume; Di: Response factor of 1 pmol biogenic amine standard; E: Protein content of sample; f: recovery factor of Internal Standard (IS area in calibration/IS area in actual). The recovery of the analysis was 86.91%.

### Statistical analysis

For the analysis of RNA level by real-time RT-PCR the ΔΔCt method was used. ΔCt is equal to the difference between a gene of interest and the average of the reference gene, ΔΔCt was calculated as ΔCt (treated) – average ΔCt (control) and fold change was determined as 2^−(ΔΔCt)^ value^48^.

For the assessment of data distribution, the Shapiro–Wilk test was used. Based on the test results, for pairwise comparisons unpaired t-test, or the non-parametric Mann–Whitney test was implemented. For the comparison of multiple study groups one-way ANOVA or Kruskal Wallis test was applied, followed by Dunnett’s and Dunn’s multiple comparison test. Correlation analysis was performed by determining the Pearson correlation coefficient. P value under 0.05 was considered significant. Statistical analyses were performed with the use of GraphPad Prism 9.

## Supplementary Information

Supplementary Information.

## Data Availability

The datasets analysed during the current study are available from the corresponding author on reasonable request.

## Abbreviations

PD: Parkinson’s disease
lncRNA: Long non-coding RNA
NEAT1: Nuclear enriched abundant transcript 1
ALS: Amyotrophic lateral sclerosis
MPTP: 1-Methyl-4-phenyl-1,2,3,6-tetrahydropyridine
MPP +: 1-Methyl-4-phenylpyridinium
SFN: Sulforaphane
HSF1: Heat shock factor 1
DA: Dopamine
5-HT: Serotonin
3-MT: 3-Methoxytyramine
DOPAC: 3,4-Dihydroxyphenylacetic acid
HVA: Homovanillic acid
5-HIAA: 5-Hydroxyindoleacetic acid
HSP: Heat shock pathway
ROS: Reactive oxygen species
MAO: Monoamine oxidase
MPDP +: 1-Methyl-4-phenyl-2,3-dihydropyridinium
DAT: Dopamine transporter
IHC: Immunohistochemistry
NA: Norepinephrine
HPLC: High-performance liquid chromatography
