# Supplementary material for: Distinct expression of NEAT1 isoforms in Parkinson’s disease models suggests different roles of the variants during the disease course
Source: Sci Rep. 2025 Apr 15;15:13027. doi: 10.1038/s41598-025-95787-0 (PMC12000440; doi:10.1038/s41598-025-95787-0)
Supplement: Supplementary file 1 — Supplementary Information. [file 41598_2025_95787_MOESM1_ESM.docx]

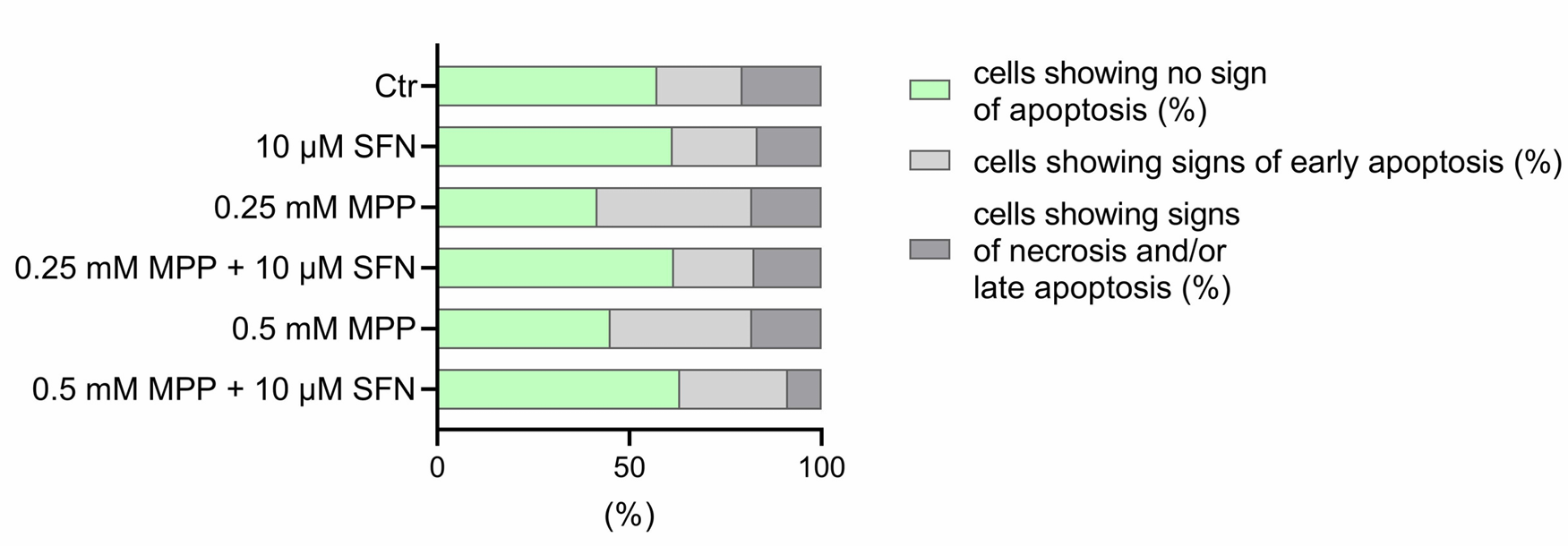


**Supplementary Figure 1.**

**Effects of SFN treatment on MPP+ toxin induced apoptosis of SH-SY5Y cells**

SFN treatment had a rescue effect on the MPP+ treatment-induced apoptosis of SH-SY5Y cells. Annexin V-FITC fluorescence-activated sorting of cells treated with MPP+ in combination with SFN for 24 hours show a smaller fraction of cells displaying signs of apoptosis than analyses of cells treated similarly but in the lack of SFN. The data shown are summary of a representative experiment.

Abbreviations: NEAT1: Nuclear Enriched Abundant Transcript 1; SFN: Sulforaphane; MPP: 1-methyl-4-phenylpyridinium; Ctr: control.


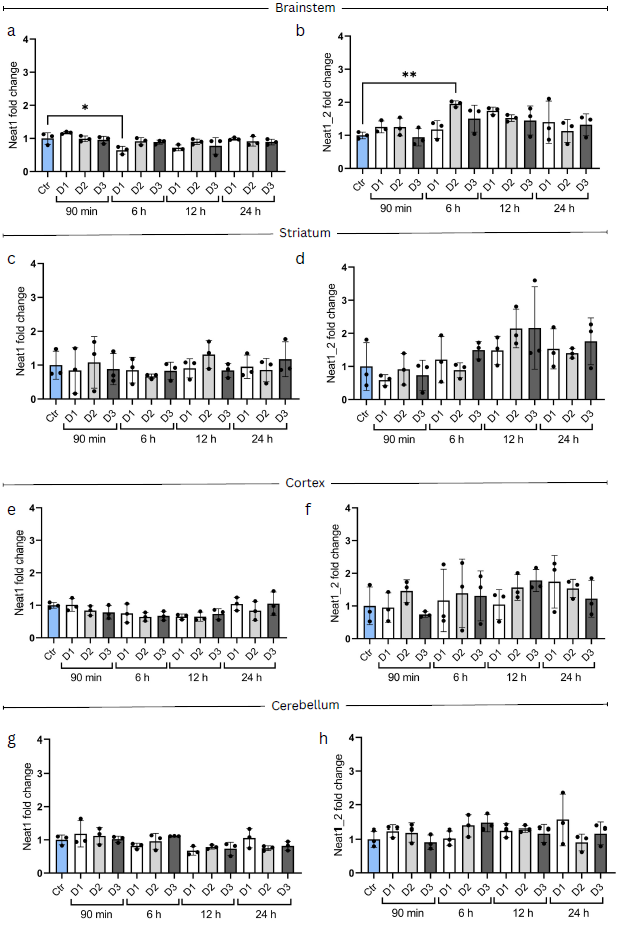


**Supplementary Figure 2.**

**Evaluation of the effect of systemically administered SFN on the expression of Neat1 in mouse brain**

Mice were treated with different doses (D1=2.5, D2=5, and D3=10 mg/kg of body weight) of SFN, and Neat1 and Neat1_2 levels were evaluated in brainstem, striatal, cortical, and cerebellar samples 1.5, 6, 12 and 24 h following treatment. Neither of the tested conditions resulted in significant increase in the level of Neat1 in any of the investigated brain regions (a, c, e, g). Similarly, no significant increase was detected in Neat1_2 levels in striatum (d), cortex (f), and cerebellum (h) samples. In brainstem samples a slight, but significant increase in Neat1_2 level was observed in animals treated with the tested higher dose at 6 h (b) (brainstem Neat1_2 expression of Ctr vs D2 treatment for 6 h: p= 0.0315).

For the assessment of data distribution the Shapiro–Wilk test was used. For graphs (b) and (c) Kruskal-Wallis test together with Dunn’s multiple comparison test was implemented. For all other graphs presented in the figure one-way ANOVA was used and for correction of multiple comparisons the Dunnett’s multiple comparison test was implemented (n=3). Values are plotted as mean±SD. Datapoints represent values detected in individual animals.

Abbreviations: D1: dose 1; D2: dose 2; D3: dose 3; Ctr: control.


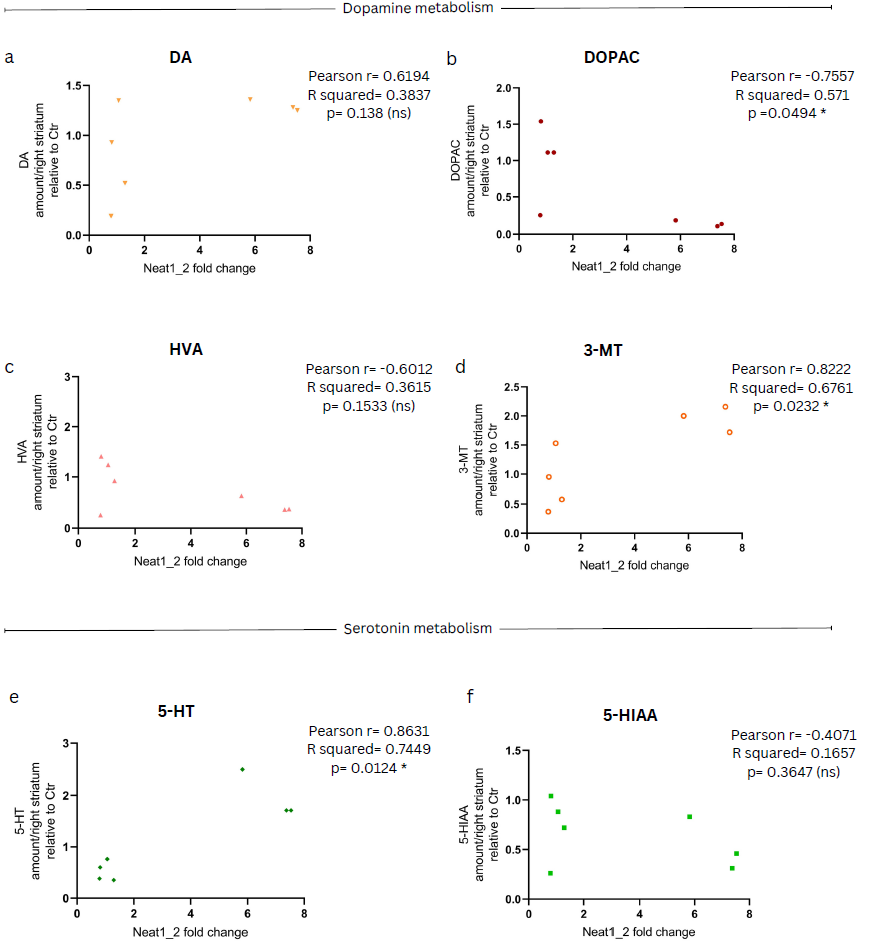


**Supplementary Figure 3.**

**Correlation analysis between Neat1_2 expression and levels of striatal DA and 5-HT metabolite levels in the PSS MPTP model**

Correlation analysis revealed no significant association between Neat1_2 expression and the levels of DA and 5-HT metabolites DA (a), HVA (c), and 5-HIAA (f), but significant negative correlation is observable between Neat1_2 and (b) DOPAC levels, and positive correlation between (d) 3-MT (e) 5-HT and the levels of the long isoform of lncRNA.

For the assessment of data distribution the Shapiro–Wilk test was used. For correlation analysis the Pearson correlation test was implemented. Datapoints represent values detected in individual animals.

Abbreviations: DA: dopamine; DOPAC: 3,4-Dihydroxyphenylacetic acid; HVA: Homovanillic acid; 3-MT: 3-Methoxytyramine; 5-HT: serotonin; 5-HIAA: 5-Hydroxyindoleacetic acid.


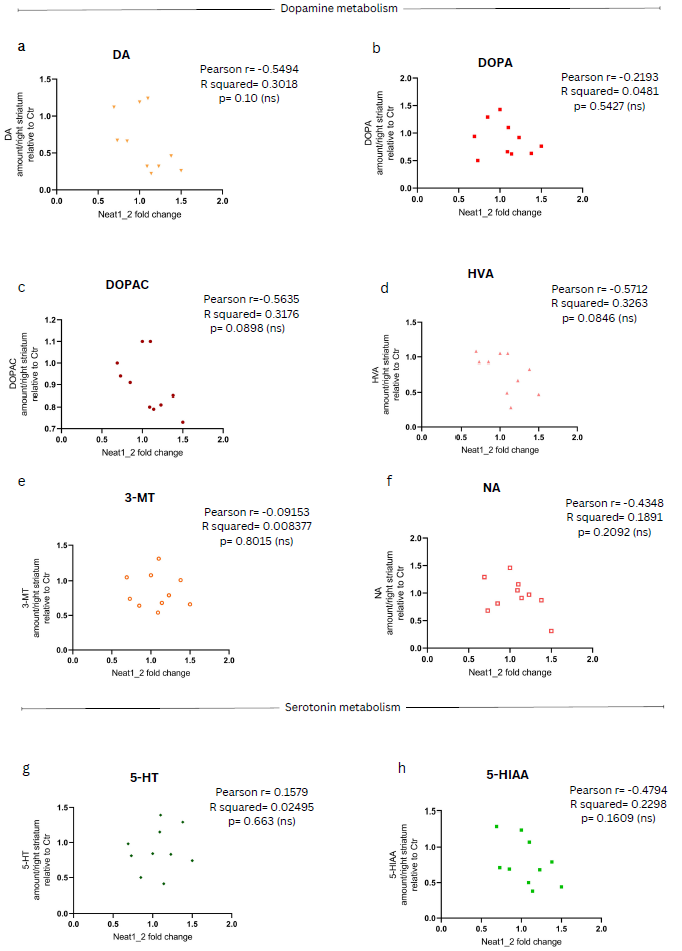


**Supplementary Figure 4.**

**Correlation analysis between Neat1_2 expression and levels of striatal DA and 5-HT metabolite levels in the ESS MPTP model**

No significant association was detectable by correlation analysis between Neat1_2 expression and the levels of any of the analysed DA (a-f) and 5-HT (g-h) metabolites.

For the assessment of data distribution the Shapiro–Wilk test was used. For correlation analysis the Pearson correlation test was implemented. Datapoints represent values detected in individual animals.

Abbreviations: NA: noradrenalin; and as in Suppl. Fig. 3.
